# Using Failure Mode, Effect and Criticality Analysis to improve safety in the cancer treatment prescription and administration process

**DOI:** 10.1186/s40545-023-00512-9

**Published:** 2023-01-19

**Authors:** Alessandra Buja, Giuseppe De Luca, Ketti Ottolitri, Elena Marchi, Francesco Paolo De Siena, Giovanni Leone, Pietro Maculan, Umberto Bolzonella, Riccardo Caberlotto, Giovanni Cappella, Giulia Grotto, Gaia Lattavo, Benedetta Sforzi, Giovanni Venturato, Anna Maria Saieva, Vincenzo Baldo

**Affiliations:** 1 Department of Cardiac, Thoracic, Vascular Sciences and Public Health, University of Padua, 35131 Padua, Italy; 2 Veneto Institute of Oncology IOV - IRCCS, Padua, Italy

**Keywords:** Patient safety, Proactive management, Chemotherapy administration, Cancer treatment

## Abstract

**Background:**

Administering cancer drugs is a high-risk process, and mistakes can have fatal consequences. Failure Mode, Effect and Criticality Analysis (FMECA) is a widely recognized method for identifying and preventing potential risks, applied in various settings, including healthcare. The aim of this study was to recognize potential failures in cancer treatment prescription and administration, with a view to enabling the adoption of measures to prevent them.

**Methods:**

This study consists of a FMECA. A team of resident doctors in public health at the University of Padua examined the cancer chemotherapy process with the support of a multidisciplinary team from the Veneto Institute of Oncology (an acknowledged comprehensive cancer center), and two other provincial hospitals. A diagram was drafted to illustrate 9 different phases of chemotherapy, from the adoption of a treatment plan to its administration, and to identify all possible failure modes. Criticality was ascertained by rating severity, frequency and likelihood of a failure being detected, using adapted versions of already published scales. Safety strategies were identified and summarized.

**Results:**

Twenty-two failure modes came to light, distributed over the various phases of the cancer treatment process, and seven of them were classified as high risk. All phases of the cancer chemotherapy process were defined as potentially critical and at least one action was identified for a single high-risk failure mode. To reduce the likelihood of the cause, or to improve the chances of a failure mode being detected, a total of 10 recommendations have been identified.

**Conclusions:**

FMECA can be useful for identifying potential failures in a process considered to be at high risk. Safety strategies were devised for each high-risk failure mode identified.

## Key points

FMECA is a proactive risk assessment tool that has proved useful in identifying potential failures in the process of prescribing and administering cancer treatments, which is a high-risk process.

The analysis evidenced 22 failures modes, distributed over the various phases of the process.

In order to reduce the likelihood of the cause, or to improve the chances of a failure mode being detected, this work identified a number of recommendations to be adopted in the administration of cancer treatment in an outpatient setting.

## Introduction

Medication errors are of particular concern because of their increasing occurrence and preventable nature. This type of errors can have fatal consequences for various reasons, from toxicity-related issues to inefficacy of the treatments [1, 2]. The European Medicine Agency defines a medication error as an unintended failure in the drug treatment process that leads to, or has the potential to lead to, harm to the patient. Mistakes in the prescription, dispensing, storage, preparation and administration of a medicine are the most common preventable causes of adverse events in medication practice, and they carry a heavy public health burden [3]. It was estimated by the National Academy of Medicine (ex-IOM) in the US that medication errors cause the death of 1 in every 131 outpatients and 1 in every 854 inpatients [4].

The impact of medication errors is such that numerous organizations, both national and international, have published recommendations in order to prevent or minimize their effects on patients, covering the various phases of the treatment process [5–8]. To address the clinical risks the Joint Commission on the Accreditation of Healthcare Organizations (JCAHO) expects healthcare institutions to conduct proactive risk management activities to identify weaknesses in their treatment prescription and administration processes, predict their possible effects, and adopt system changes to minimize the potential harm to patients [9, 10]. One such activity involves conducting a failure mode and effects analysis (FMEA) [11]. This proactive risk assessment tool is used to identify potential vulnerabilities in complex, high-risk processes, and to generate remedial actions before they can result in adverse events. FMEA is also increasingly used to proactively assess and improve the safety of complex healthcare processes such as drug administration and blood transfusion [12]. The Institute for Safe Medication Practices has been recommending the use of FMEA to prevent medication errors since the mid-1990s [13].

A previous editorial piece stressed that relatively little time and money have been devoted to the safety and quality of cancer care, including attention to reducing medication-related errors, although the importance of these issues has been acknowledged, and although a tremendous amount of cancer research is performed every year [14]. Amidst the growing demand for cancer care, combined with the complexity of the disease and its treatment, a shrinking workforce has given rise to a crisis in cancer care delivery. In recent years, cancer patients’ care and treatment have been shifted increasingly towards more outpatient services, fewer hospital admissions, and shorter hospital stays [15, 16]. Today, most cancer treatments are provided in outpatient settings [17]. Treating cancer is a high-risk process and the second most common situation in which fatal medication errors occur in US [18]. This has also been demonstrated for the Italian context, where antineoplastic and immunomodulating agents are the second most frequently implicate drug category in fatal adverse drug reactions [19, 20].

The complexity of chemotherapeutic formulations and the narrow therapeutic index of many drugs make the process susceptible to critical errors. Cancer treatments have also become increasingly lengthy, involving different health professionals and settings, and different medical disciplines, and this adds to the risk of errors occurring. In this regard, the United States Pharmacopeia (USP) analyzed the distribution of the different types of errors in cancer treatment (1998–2003) showing that 25% of the errors were attributable to improper dose/quantity, 20.4% to a prescription error, 18.6% to omission error, 12.5% to wrong time, less frequently to unauthorized drug, to wrong preparation of drug, to Extra dose, to wrong administration technique, to wrong patient, to wrong route, or wrong dosage form [21].

The importance of the problem has now been recognized and—in Italy, for instance—it has been addressed by the Ministry of Health, which has issued specific recommendations concerning the prescription and administration of cancer treatments [22].

In such a setting, it can be extremely useful to apply Failure Modes Effect and Criticality Analysis (FMECA) to assess these processes and improve patient safety. The purpose of this study was to conduct a prospective, systematic analysis of the various phases comprising the process of chemotherapy prescription and administration, applying FMECA methodology to identify possible errors and enable the adoption of measures to prevent their occurrence.

## Methods

The FMECA method was applied to the cancer medication prescription and administration process in order to highlighting possible errors at different stages of the entire process and prioritizing the subsequent interventions. The analysis was conducted from April to June 2022, considering the process for prescribing and administering cancer therapies at outpatient clinics managed by the Veneto Institute of Oncology-IRCCS (IOV-IRCCS) (a comprehensive cancer center is recognized by the Organization of European Cancer Institutes that serves as a regional hub for the diagnosis and treatment of oncological diseases), and at two other medical oncology units at two provincial hospitals in the Veneto region of Italy (the Ospedale dell’Angelo in Mestre [Venice] and the Ospedale “San Bortolo” in Vicenza).

After an introductory session held with a team of experts to explain the characteristics of FMECA, the prescription and administration of cancer therapies was chosen as a high-risk process to be investigated. The multidisciplinary team consisted of public health residents from the University of Padua working at different hospital administrations in the Veneto region, together with public health specialists, risk managers and oncology nurses from the outpatient clinics of the units involved.

The FMECA process included six steps, as listed in Table 1.

**Table 1:** General steps for conducting a Failure Mode, Effect and Criticality Analysis

Step	Description
1	Choose a process to investigate
2	Form a multidisciplinary team
3	Map out the process, including each step
4	Calculate a risk priority for each step in the process
5	Select an area for improvement based on the risk priority calculated
6	Implement actions and obtain outcome measures

**Fig. 1:**
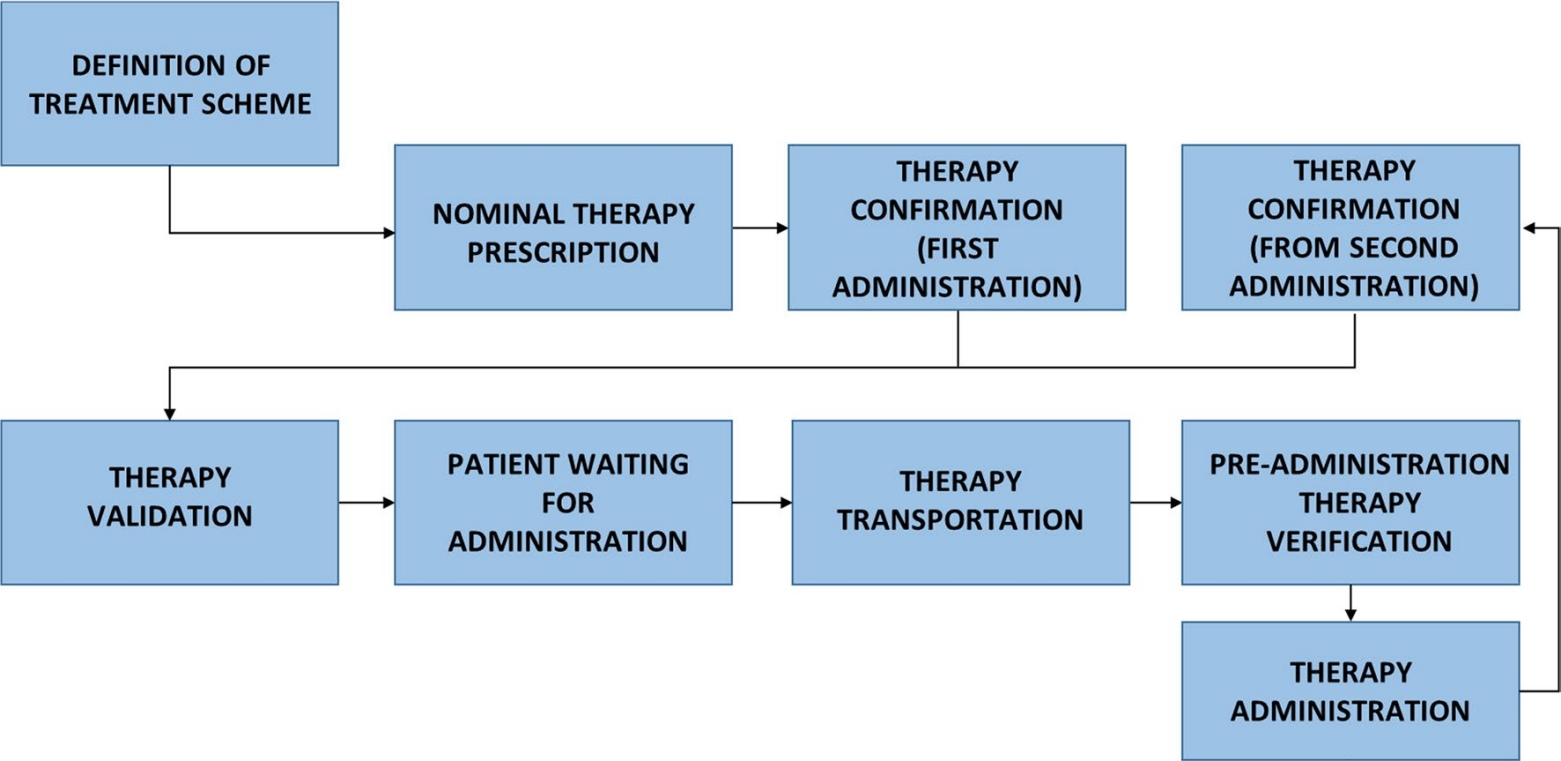
Phases of the drug administration process

To visualize the process and subprocesses to examine, a diagram was developed that shows the various steps involved in prescribing and administering cancer therapies (Fig. 1).

The possible failures in each subprocess were identified using brainstorming techniques based on the subjective experience of the professionals involved and a study of the clinical units’ operating instructions covering the whole process, which were made available to the team.

Analyzing the process enabled failure modes and critical issues to be identified by classifying them in terms of: frequency of occurrence (probability of the event occurring); detectability (probability of the error being discovered before it affects the patient); severity (effect of the failure on the patient). Table 2 shows the rating scale used in this study, which is adapted from those used in previously published studies [23, 24]. Three team members scored each possible failure on frequency of occurrence, detectability, and severity.

**Table 2:** Ratings for frequency of occurrence, detectability and severity

	Rating
*Frequency of occurrence of failure mode*	
Once a year	1
Once a month	2
Once a week	3
Once a day	4
Several times a day	5
*Detectability of failure mod*e	
≥ 90%	1
≥ 75%	2
≥ 50%	3
≥ 25%	4
≥ 10%	5
0–9%	6
*Severity of the effect of the failure mode*	
Slight annoyance: may affect the cancer treatment system	1
Moderate system problem: may affect the patient	2
Major system problem: may affect the patient	3
Minor injury	4
Major injury	5
Terminal injury or death	6

The Risk Priority Number (RPN) was then calculated to numerically assess the risk assigned to each failure mode by multiplying the numerical scores for the three items (RPN = severity × occurrence × detectability). The median values of the RPNs for the failure modes identified were used to obtain a final score. The ranges of the RPNs are also reported. In the context of a FMECA, the RPN is therefore a numerical estimate of the risk attributed to a process, or a step in a process (or, in other words, an indication of its criticality). It identifies the elements most likely to contribute to medically serious failures. The maximum RPN is 180, and it has been assumed that any RPN > 20 identifies a high-risk failure. This is a conventional value, corresponding to the 75th percentile, employed to prioritize failures although all failures should be addressed (more or less promptly).

The team considered the criticality of each failure mode, and decided whether the risk was acceptable or improvements were needed. Failures with the highest scores were classified as high risk and identified as priority areas where safety strategies needed to be implemented.

## Results

The analysis pointed to 22 possible failures distributed over the various phases of the treatment prescription and administration process (Fig. 1), and 7 of them were classified as high risk.

The phases with the largest number of possible failures were “Confirming therapy (from second administration onwards)” (2), and “Checking therapy prior to administration” (2).

The RPNs (Table 3) ranged from 6 to 30. The most important failures with the highest RPNs were found in the “Validating therapy” and “Checking therapy prior to administration” phases. They included, for instance “Error in validating previously prescribed and confirmed therapy”, “Error in identifying the right patient”, and “Error in identifying the therapy to administer to the patient”.

**Table 3:** Possible failures of the treatment prescription and administration process, ranked by RPN in each phase with sum of criticality indices

Phases and possible failure modes	RPNs (medians and ranges)
*Defining treatment plan*	26
Errors or failures in the entry of preset treatment plans	10 (5–10)
Failure or delay in updating preset treatment patterns	8 (4–10)
Failure to notify treatment providers of updates or new preset treatment patterns available	8 (4–10)
*Nominal therapy prescription*	64
Failure to prescribe premedication tailored to the type of patient (e.g., allergy history)	24 (12–36)
Failure to prescribe or incorrect prescription of ancillary/supportive therapy (antiemetics, PPIs, etc.)	16 (16–20)
Prescribing physician's error in filling out required forms	12 (10–20)
Failure to evaluate results of blood tests mandatory for the administration of the treatment	8 (2–8)
Therapy prescription impossible due to IT malfunctions (e.g., computer-based medical records)	4 (4–4)
Confirming therapy (first administration)	12
Incorrect/incomplete evaluation of results of blood tests and/or other tests required for the established treatment plan	12 (10–12)
Confirming therapy (from second administration onwards)	40
Incorrect/incomplete evaluation of results of blood tests and/or other tests required for the established treatment plan	20 (10–32)
Incomplete evaluation of toxicities encountered following administration of chemotherapy, failure to modify treatment dosage, failure to administer ancillary/supportive therapy (steroids, antiemetics, PPIs, etc.)	20 (20–42)
*Validating therapy*	30
Error in validating previously prescribed and confirmed therapy	30 (10–32)
*Patient waiting for treatment to be administered*	28
Overcrowded waiting areas	16 (2–24)
Patient fainting	12 (10–18)
*Transportation of drugs*	30
Damage to preparations in transit	30 (10–40)
*Checking therapy prior to administration*	60
Error in identifying the right patient	30 (10–40)
Error in identifying the therapy to administer to the patient	30 (10–50)
*Administering therapy*	46
Delay in drug administration	16 (10–24)
Damage due to chemotherapy extravasation	10 (8–16)
Adverse reactions due to noncompliance with administration schedules	8 (8–10)
Adverse reactions due to errors in the sequence of administration of infusion bags, or to failure to administer ancillary therapies	6 (6–9)
Failure to act or delay in response to pump alarm or patient’s call	6 (6–6)

The sum of criticality indexes was 26 for “Defining treatment plan” phase, 64 for “Nominal therapy prescription” phase”, 12 for “Confirming therapy (First administration)” phase, 40 for “Confirming therapy (from second administration onwards)” phase, 30 for “Validating therapy” phase, 28 for “Patient waiting for treatment to be administered” phase, 30 for “Transportation of drugs” phase, 60 for “Checking therapy prior to administration” phase, and 46 for “Administering therapy” phase.

For the potentially high-risk failure modes, at least one recommended action was identified with a view to reducing their occurrence or improving their detection. This resulted in the 10 recommendations listed in Table 4.

**Table 4:** Actions recommended to improve the cancer treatment prescription and administration process

Process phase	Recommended actions
Nominal therapy prescription	Verbally request that patients confirm their personal details, weight/height, and the name of their medication
	System flag required for changes to the mode of administration for a given treatment (compared with standard procedure). System flag for therapies requiring a CVC
	Pharmacist should check mode of administration and dosage when different from standard procedure before validating a therapy
Confirming therapy (from second administration onwards)	System warning in event of abnormal exam values being input
	Method (e.g., boxes to tick) for checking that specific blood tests required by the treatment plan have been performed
	Mandatory check (e.g., boxes to tick) on whether any adverse reactions occurred after the previous administration
	System warning in event of no ancillary therapy or premedication. Therapy confirmation check list
Transportations of drugs	Medications must be transported in a closed, leak-proof plastic bag and be carried in a rigid, shock-resistant, leak-proof container. If drugs are not administered immediately, they should be stored at room temperature or refrigerated to avoid deterioration
Validating therapy	Automated system warnings in event of any deviations from the protocol envisaged in the treatment plan
Checking therapy prior to administration	Barcode cross-check on bags, labels and medical records (therapy prescribed / therapy prepared)

## Discussion

Each phase of the cancer chemotherapy process is potentially critical. The FMECA conducted in present study revealed more than 20 failure modes distributed over the various phases of the process, one in three of them classified as high risk. At least one action for each of the high-risk failure modes was identified as capable of limiting these failures or improving their detection, for a total of 10 recommendations.

Consistent with the literature, [25] our results indicate that the "nominal therapy prescription" phase is the one most at risk because it is characterized by several possibilities for error. In fact, the safety management of this particular phase has developed specific tools to reduce the occurrence and increase the detectability of these various errors, ultimately helping to decrease their RPN, e.g., a computerized physician order entry (CPOE) system, a technology which positively affects the safety of drug administration, was introduced in the hospitals where the study took place. Using CPOE avoids errors due to poor handwriting or incorrect transcription [26]. The technology also includes functionalities such as drug dosage support, and alerts about harmful interactions, thereby contributing to further reducing the potential for errors [27].

The analysis revealed other possible failures in the “Nominal therapy prescription phase”, however. The most critical is “Failure to prescribe premedication tailored to the type of patient (e.g., allergy history)”, which could result in anaphylaxis during the therapy’s administration. This is coherent with the literature, which has already shown that allergic reactions and adverse drug reactions are an important source of medication-related adverse events [28]. Such failures were recognized as being associated with “Incorrect/incomplete evaluation of results of blood tests and/or other tests required for the established treatment plan” and “Incomplete evaluation of toxicities encountered following administration of chemotherapy, failure to modify treatment dosage, or failure to administer ancillary/supportive therapy (steroids, antiemetics, PPIs, etc.)”. In this context, implementing clinical decision support systems in CPOE may raise safety levels by giving physicians default values for doses and administration routes in the drug prescription phase [29]. Such systems may also offer additional sophisticated drug safety features, such as checking for drug allergies or drug–drug interactions, providing reminders for appropriate laboratory monitoring, or even suggesting appropriate prescription based on patient-specific factors [30, 31].

In our analysis, the “Checking therapy prior to administration” phase resulted the second most critical phase of the process. Two potential failure modes relating to this phase namely “Error in identifying the right patient” and “Error in identifying the therapy to administer to the patient" were well described in other studies and emerged as relevant in our analysis too [32–34]. In fact, the misidentification of patients is still a fundamental patient safety issue, and its eradication would be a major step in improving healthcare quality. Among the countermeasures applied in the setting examined here, verbally identifying patients by asking them for their full name and date of birth is one of the first, basic remedial actions proposed. Other strategies already mentioned in the literature include using cross-checks with barcode label/reader systems to match patients and therapies, but their value in the outpatient setting is still being debated.

Although the present analysis showed no critical failures in the "therapy administration" phase, this one is characterized by a large number of potential failures, making it the third most critical. This is in line with the literature, which indicates the administration phase as one of the most error-prone phases of medication process [35]. In this context, the technology-controlled administration of drugs using a smart pump is one of the ways to reduce errors in this phase of the chemotherapy delivery process. This tool has become standard practice in hospitals when administering critical fluids (such as those containing medications) to patients and it has been shown to prevent 5% of errors in intravenous chemotherapy administration [36, 37]. Compared with the manual administration of fluids, smart pumps offer the advantage of a controlled delivery: they can administer fluids in small volumes or at precisely programmed rates or intervals.

Our analysis showed that “Transportation of drugs” phase must be adequately considered because of the critical risk represented by possible damage to preparations during the transit from the pharmacy to the outpatient clinic. Indeed, the principle of guaranteeing the delivery of therapy to “the right place at the right time for the right person” requires a safe and reliable method of transporting drugs to increase the traceability and reliability of the drug delivery process [38]. International practice guidelines recommend that medications must be transported in a closed, leak-proof plastic bag from the pharmacy and be carried in a rigid, shock-resistant, leak-proof container made of a material that can be easily cleaned and decontaminated. In addition, if drugs are not administered immediately, they should be stored at room temperature or refrigerated to avoid deterioration that could reduce their efficacy or safety [39, 40].

As underscored in a previous work, the risk of transmission of airborne diseases (e.g., SARS-CoV-2) as a result of lengthy stays in potentially overcrowded spaces like outpatient waiting rooms deserves to be considered separately [23]. Although the likelihood and severity of this failure mode is hard to estimate, it should still be addressed because of the potential consequences of such infections for patients, especially those experiencing therapy-induced neutropenia. There is still not enough research and reporting on protocols for the care of cancer outpatients during outbreaks of infectious diseases. For the time being, frequent hand washing, social distancing, wearing medical masks, and—where necessary—routine screening have been proposed as standard practices that all outpatient cancer units should enforce in preparation for future pandemics [41].

### Limitations

The FMECA methodology suffers from several limitations, the main one being an inevitable subjectivity in the selection of failure modes and the calculation of criticality indices. To minimize this drawback in the present study, explicit criteria were established for assessing the frequency, detectability and severity of the failure modes envisaged, and each failure was discussed by all team members.

It should also be noted that, although the failures discussed here may be applicable elsewhere, the diversity of healthcare services and differences in their structural features (e.g., their use of information technology) make any definition of RPNs context-specific. It would consequently be useful to interview personnel employed in other countries with a view to developing a more generalizable model.

## Conclusions

In conclusion, the present findings show that FMECA can be a helpful tool for improving healthcare quality and reducing errors in the treatment of patients. It proved extremely useful in revealing where improvements could be made in the cancer drug therapy prescription and administration process.

## Data Availability

The datasets used and/or analyzed during the current study are available from the corresponding author on reasonable request.
